# *EuroSCORE* II and the importance of a local model, InsCor

**DOI:** 10.1186/1749-8090-8-S1-O115

**Published:** 2013-09-11

**Authors:** L Lisboa, O Mejia, L Moreira, L Dallan, PMA Pomerantzeff, L Dallan, M Massoti, D Vianna, F Jatene

**Affiliations:** 1 Cardiovascular Surgery Department, Heart Institute (InCor), Hospital das Clínicas da Faculdade de Medicina da Universidade de São Paulo, São Paulo, Brazil

## Background

The most widely used model for predicting mortality in cardiac surgery was recently remodeled, but the doubts regarding its methodology and development have been reported.

The aim of this study was to evaluate the performance of the EuroSCORE II to predict mortality in patients undergoing coronary artery bypass grafts or valve surgery at our institution.

## Methods

One thousand consecutive patients operated on coronary artery bypass grafts or valve surgery, between October 2008 and July 2009, were analyzed. The outcome of interest was in-hospital mortality. Calibration was performed by correlation between observed and expected mortality by Hosmer Lemeshow. Discrimination was calculated by the area under the ROC curve. The performance of the EuroSCORE II was compared with the EuroSCORE and InsCor (local model).

## Results

In calibration, the Hosmer Lemeshow test was inappropriate for the EuroSCORE II (P = 0.0003) and good for the EuroSCORE (P = 0.593) and InsCor (P = 0.184). However, the discrimination, the area under the ROC curve for EuroSCORE II was 0.81 [95% CI (.76 to .85), P <0.001], for the EuroSCORE was 0.81 [95% CI (0.77 to 0.86), P <0.001] and for InsCor was 0.79 [95% CI (0.74-0.83), P <0.001] showing up properly for all.

## Conclusion

The EuroSCORE II became more complex and resemblance to the international literature poorly calibrated to predict mortality in patients undergoing coronary artery bypass grafts or valve surgery at our institution. This emphasizes the importance of the local model.

